# Explorative Study on the Use of Curauá Reinforced Polypropylene Composites for the Automotive Industry

**DOI:** 10.3390/ma12244185

**Published:** 2019-12-12

**Authors:** Marc Delgado-Aguilar, Quim Tarrés, María de Fátima V. Marques, Francesc X. Espinach, Fernando Julián, Pere Mutjé, Fabiola Vilaseca

**Affiliations:** 1 LEPAMAP Group, Department of Chemical Engineering, University of Girona, 17003 Girona, Spain; m.delgado@udg.edu (M.D.-A.); joaquimagusti.tarres@udg.edu (Q.T.);; 2 Instituto de Macromoléculas, Universidad Federal do Rio de Janeiro, Rio de Janeiro CEP 21941-598, Brasil; fmarques@ima.ufrj.br; 3 Design, Development and Product Innovation, Dept. of Organization, Business, University of Girona, 17003 Girona, Spain; francisco.espinach@udg.edu (F.X.E.); fernando.julian@udg.edu (F.J.); 4 Department of Fibre and Polymer Technology, KTH Royal Institute of Technology, SE-10044 Stockholm, Sweden

**Keywords:** automotive industry, natural fiber, composites, polypropylene, stiffness, curauá fibers

## Abstract

The automotive industry is under a growing volume of regulations regarding environmental impact and component recycling. Nowadays, glass fiber-based composites are commodities in the automotive industry, but show limitations when recycled. Thus, attention is being devoted to alternative reinforcements like natural fibers. Curauá (Curacao, *Ananas erectifolius*) is reported in the literature as a promising source of natural fiber prone to be used as composite reinforcement. Nonetheless, one important challenge is to obtain properly dispersed materials, especially when the percentages of reinforcements are higher than 30 wt %. In this work, composite materials with curauá fiber contents ranging from 20 wt % to 50 wt % showed a linear positive evolution of its tensile strength and Young’s modulus against reinforcement content. This is an indication of good reinforcement dispersion and of favorable stress transfer at the fiber-matrix interphase. A car door handle was used as a test case to assess the suitability of curauá-based composites to replace glass fiber-reinforced composites. The mechanical analysis and a preliminary lifecycle analysis are performed to prove such ability.

## 1. Introduction

The automotive industry is under a growing volume of regulations, related to safety and security, emissions, recyclability and other aspects [1]. These legislations can change noticeably from one country to the next, but are usually in line with the policies and objectives of the governments and the society. Sometimes, these legislations seem to be contradictory. As an example, increasing the security of the vehicles demands the incorporation of elements that can increase its weight. It is known that around 50% of the fuel consumption of an automobile depends upon its mass [2]. Furthermore, lifecycle analysis in the car industry revealed that the environmental impact of a vehicle mainly occurs in its use phase [2,3]. In order to overcome these apparent conflicts between security and fuel consumption, and in parallel with other solutions, the automotive industry has devoted great efforts in light-weighting its components [2,4].

The light-weighting of a vehicle can be achieved by design changes, modification of the construction processes or the use of light engineering materials [2,5]. In addition to the above-mentioned regulations, the automotive industry has also to fulfill new regulations related to the end of life of the vehicles. Such regulations, like the European Union (EU) End of Life Vehicle directive, restrict the amount of vehicle that can be landfilled at its disposal. This directive enforces recovering and reusing at least 95% of the vehicle [6,7].

The automotive industry has explored alternatives to steel that provide the required mechanical properties while being lighter. These materials include metals like aluminum or magnesium, polymers like polyolefin, or composite materials like glass fiber or natural fiber-reinforced polymers. Moreover, alternative materials must show technical, economic and environmental performances [2].

The use of natural fiber-reinforced polymers has caught the attention of automotive industry, due to certain reasons. On the one hand, natural fibers come from renewable resources, locally available and comparatively cheaper than mineral fibers [8,9,10]. On the other hand, natural fibers are lighter than glass fibers, and thus, natural fiber-reinforced materials tend to show higher specific properties [11]. Nowadays, the use of natural fiber-reinforced composites has increased [9]. These materials are used for globe boxes, door panels, seat coverings, seat surfaces, trunk panels, trunk floors, spare tire covers, insulation, headliners, or dashboards [9,12]. The use of natural fiber-reinforced composites has been limited to non-structural or semi-structural purposes, and usually as an alternative to glass fiber-reinforced materials [9,12,13]. The most common natural fibers used as reinforcement in the automotive industry are jute, flax, sisal, cotton, wood abaca and kenaf [9,13,14]. The studies on the use of curauá fibers as reinforcement in the automotive industry, to the best knowledge of the authors, are scarce [6].

Curauá is a Bromeliaceae (*Ananás erectifolius*) common to the Amazonas region. Curauá is one of the natural fibers with more potential to be used as composite reinforcement [6,15,16]. Actually, these fibers are being used in the textile and automotive industries [6,17]. Furthermore, curauà fiber is odorless, enabling its use for car interior parts [6].

The use of natural fibers as reinforcement in composites has been extensively reported in the literature [8,9,10,18,19,20,21,22]. Nonetheless, the use of lignocellulosic reinforcements limits the range of polymers to be used as a matrix, mainly because cellulose starts to degrade fast when exposed to temperatures beyond 200 °C [23].

The mechanical properties of a fibers-reinforced composite are mainly affected by the nature of its phases and its percentages, its compatibility, as the ability to create strong interphases between the reinforcement and the matrix, the morphology of the reinforcements and its dispersion and its main orientation against the loads. While all the above-mentioned aspects have importance, a composite will always fail due to its feeblest phase, usually the interphase [13,24,25]. Obtaining strong interphases in natural fiber-reinforced composites is hindered by the different natures of these phases. Matrices used to be hydrophobic, and natural fibers hydrophilic, hindering a correct wetting of the reinforcements and disabling the creation of chemical bonding [26,27]. Thus, a great effort has been devoted to overcome this problem. The methods that have showed better results were based on fiber treatments or the use of coupling agents [28,29,30,31]. These methods enable the correct wetting of the reinforcements and the creation of hydrogen bonds, obtaining strong interphases. It must be mentioned that the use of matrices functionalized with maleic anhydride have reported excellent results in the case of polyolefin [25,32,33].

In the case of curauá-reinforced composites, the matrices that are mainly used are polyolefin, like polypropylene and high-density polyethylene, in some cases biobased. Castro et al. produced biobased composites by reinforcing a biopolyethylene with curauá fibers [15]. These researchers used a liquid hydroxylated polybutadiene as a coupling agent. The produced composites showed decreasing flexural strengths with increasing reinforcement contents. This is usually a sign of a weak interphase. In another research, the authors used castor and canola oils as compatibilizers in curauá-reinforced bio-polyethylene composites [34]. Here the authors observed increases on the flexural strengths of the composites due to the presence of the coupling agents. Nonetheless, the flexural strength tends to decrease with increasing percentages of reinforcement.

The authors blame this on a poor dispersion of the reinforcements in the composite. Nacas et al. produced curauá-reinforced polypropylene composites [35]. These researchers used raw and fibrillated reinforcements and maleic anhydride as their coupling agent. The materials showed noticeable increases of its tensile strength when fibrillated reinforcements and coupling agents were used in the formulation of the composites. The study was limited to 20 wt % reinforcement contents. Mano et al. studied the effect of processing conditions on the mechanical properties of curauá-reinforced polypropylene and polyethylene composites [36]. These authors did not use any coupling agent. The research stated the impact of the morphology of the reinforcements on the mechanical properties of the composites. The reinforcements are exposed to attrition during composite preparation and experience noticeable length shortening. Thus, the processes that prevented the shortening of the reinforcements allowed the obtaining of composites with higher mechanical properties. Another research obtained uncoupled curauá-reinforced polypropylene composites [37]. These materials showed increases of their flexural strengths against reinforcement contents. The maximum content of curauá was limited to 20 wt %, possibly to avoid dispersion problems. The literature shows the importance of using a coupling agent to obtain strong interphases. There is also an unsolved bad dispersion of the fibers for percentages of curauá higher than 20 wt %.

In this work, composites based on a polypropylene matrix reinforced with curauá fibers were formulated and prepared. The composites added 20 wt %, 30 wt %, 40 wt % and 50 wt % of reinforcement to assess the effect of these percentages over the tensile strength, Young’s modulus and strain at the break of the composites. In order to obtain strong interphases, the materials added a 6 wt % against the reinforcement content of the coupling agent. The results are compared with glass fibers-reinforced composites. The tensile strength of the composites shows a linear increase up to 50 wt % cuarauá contents.

A door car handle was modeled, and a finite element analysis was used to assess the potential use of the studied composites. The results are presented and compared with other potential materials, in terms of specific properties and component lightweighting.

Finally, a preliminary lifecycle analysis was performed to assess the environmental impact of the components depending on the materials they are made of.

The main objective of the research is showing the potential of curauá fibers as polyolefin reinforcement. An automotive component has been chosen due to the global impact of such industry and its innovative nature. The results show the advantages of this natural reinforcement in front of glass fiber, in terms of environmental impact, mechanical properties and the possibility of obtaining good fiber dispersions and strong interphases at high fiber contents.

## 2. Materials and Methods

### 2.1. Materials

A polypropylene (PP) homopolymer with trademark Isplen PP099 G2M by REPSOL YPF (Tarragona, Spain) was used as our matrix. This polymer has a 0.905 g/cm^3^ density and a 55 g/10min melt flow index (230 °C, 2.16 kg). This melt flow index makes the polymer especially suitable for mold injection.

A polypropylene functionalized with maleic anhydride (MAPP), with trademark Epolene G3015 by Eastman Chemical Products (San Roque, Spain) was used as the coupling agent. The MAPP has a 15 mg KOH/g acid number as a 24800Da atomic mass.

Cuarauá fibers were obtained from the Instituto de Macromoléculas Professora Eloisa Mano Universidade Federal do Rio de Janeiro (Rio de Janeiro, Brazil). Glass fiber (GF) by Vetrotex (Chamberley, France) was provided by Maben, S.L. (Banyoles, Spain).

Other reactants used during fiber treatment were: Sodium hydroxide (NaOH) by Merck, KGaA (Darmstadt, Germany) and anthraquinone by Basf AG (Tarragona, Spain). The reactants were used as received, without any further purification.

### 2.2. Fiber Treatment

The fibers were provided as long strands, unable to be used for mold injection. Curauá strands were chopped in a knives mill and then sorted, to obtain fibers with a mean length of 2 mm. Next, the fibers were defibrated in a Sprout-Waldron equipment. The process took place at room temperature and under aqueous conditions, obtaining a curauá mechanical pulp (CF). The process rendered a 99% yield with respect to the raw strands. The CF showed a density of 1.44 g/cm^3^.

### 2.3. Composite Preparation

The composites were formulated with 20 wt %, 30 wt %, 40 wt %, 50 wt % and 60 wt % CF contents. All the composites added a 6 wt % of MAPP, against CF content. The percentage of MAPP was based on previous works and had the objective of maximizing the tensile strength of the composites [26]. MAPP was added with the PP. Composites were prepared in a kinetic internal mixer by Gelimat® (Ramsey, USA). The process took place at 3000 rpm and lasted approximately 2 min, until a 210 °C discharge temperature was reached. Although compounding decreases the mean length of the reinforcements due to attrition, Gelimat® mixers have less impact on the mean length of the reinforcements than other equipment does, and obtains reinforcements with better aspect ratios [38].

GF-based composites were prepared in a Brabender® plastograph mixer (Duisburg, Germany). The equipment was operated at 20 rpm during 10 min and at 180 °C. Coupled (GFe) and uncoupled (GFs) composites were prepared. In the case of the coupled composites, the coupling agent was added with the PP.

Before its use, the materials were pelletized in a knife mill and stored at 80 °C during at least 24 h.

### 2.4. Specimen Obtention

Standard dog-bone specimens were mold injected. The equipment was a Meteor 40 injection mold machine by Mateu & Solé (Barcelona, Spain). At least 10 valid specimens for all composite formulations were obtained. The temperature profile was 175, 175 and 190 °C for the three heating areas. The last one corresponds with the injection nozzle. First and second pressures were 120 and 37.5 kgf/cm^2^, respectively.

### 2.5. Tensile Testing

The specimens were stored in a Dycometal (Barcelona, Spain) climatic chamber at 23 °C and 50% relative humidity the 48 h before the tensile test, in agreement with ASTM D638 and ASTM D618 [39,40]. The specimens were tensile tested in an Instron 112 universal machine (Norwood, MA, USA). The machine is equipped with a 5 kN load cell and was operated at 2 mm/min. The results were the mean of at least five measurements.

### 2.6. Car Door Handle Modelling and Analysis

The original component was measured in a Mitutoyo Crysta Apex 544 coordinate measuring machine (Elgoibar, Spain). These measures were used to obtain a digital mockup with SolidWorks^®^ by Dassault Systemes (Vélizy-Villacoublay, France).

The interface conditions were obtained from the literature [13]. Use conditions were based on a normal use of a car door handle. A finite element analysis was performed by using the Simulation 2017 x64 SP3.0 module of Solid Works. The preliminary life cycle analysis (LCA) was performed with the sustainability module of Solid Works.

## 3. Results and Discussion

### 3.1. Tensile Properties of Curauá Reinforced Polypropylene Composites

The composites were submitted to tensile test to obtain its tensile strength (σ_t_^C^), Young’s modulus (E_t_^C^) and strain at break (ε_t_^C^). Table 1 shows the obtained values. In the table, V^F^ and ρC account for the reinforcement volume fraction and the composite density, respectively.

**Figure 1:**
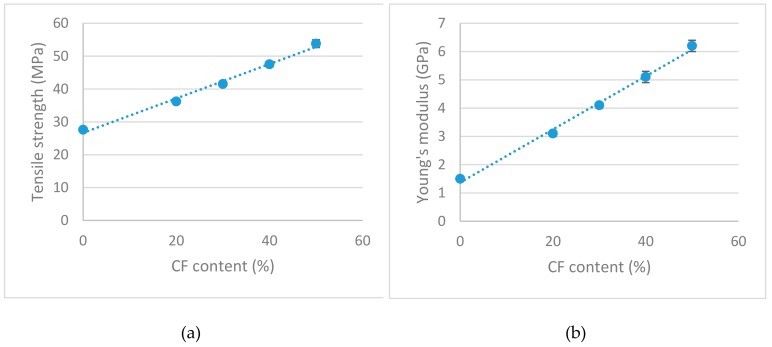
Evolution of the tensile properties of the composites against reinforcement contents: (**a**) Tensile strength; (**b**) Young’s modulus.

The results show a sustained increase of the tensile strength of the composites against CF contents. The composites increased the tensile strength of the matrix 31%, 50%, 72%, 95% and 121% by adding 20 wt %, 30 wt %, 40 wt % and 50 wt % of CF, respectively. Moreover, Figure 1a shows that the evolution of the tensile strength is also linear against CF content. The literature indicates that such behavior is possible when a proper dispersion of the reinforcements inside the composite and a strong interphase between the fibers and the matrix is also present [41].

In the case of the Young’s modulus of the composites, a sustained and linear increase against CF content was also observed (Figure 1b). The Young’s moduli of the composites increased noticeably with the CF contents. The composites increased the modulus of the matrix by 106%, 173%, 240%, 313% and 393% for the composites with 20 wt %, 30 wt %, 40 wt % and 50 wt % contents, respectively. It is known that the Young’s modulus of a composite is little affected by the strength of the interphase, but strongly affected by the dispersion of the fibers [42]. Thus, the results back the hypothesis of a proper dispersion of the fibers, but the use of a technique that permits a direct measurement of the fiber dispersion is needed to fully back such a hypothesis. Notwithstanding, a micromechanical analysis is needed to assess the strength of the interphase between CF and PP.

The elongation at break of the composites decreased as a direct consequence of the higher percentages of reinforcement, a fragile phase. Thus, although the tensile strength increased, the composites were unable to sustain high elongations.

The tensile properties of the composites are similar to other strand-reinforced composites like hemp- or jute-reinforced PP [26,43]. The results were higher than those obtained by reinforcing the same matrix with wood fibers [38,42,44]. Nonetheless, the composites that are more used in the automotive industry are GF-based. Thus, in order to assess the possibilities of using CF-based composites in the automotive industry, its properties must be compared to GF-based materials. Table 2 shows the tensile properties of GF-reinforced PP composites. The table shows the results obtained for uncoupled (GFs) and coupled (GFe) composites [38,44,45].

GF-based composites were prepared with the same matrix and the same equipment than CF-based materials, in order to discard the effect of such parameters on the tensile properties of the materials. The content of GF was limited to a maximum of 30%, because on the one hand, this is the standard in the industry, yet on the other hand, GF is highly affected by attrition during compounding. These phenomena increase with the amount of reinforcement and they reduce the length of GF [46].

The differences between the coupled and uncoupled GF composites were noticeable. Both kinds of composites increased the tensile strength of the matrix, but while uncoupled composites increased the tensile strength of the matrix by an 85% and a 112% for 20 wt % and 30 wt % GF contents, respectively, the coupled composites increased 145% and 188% for the same GF contents. This shows the effect of a strong interphase on the tensile properties of a composite. In the case of the Young’s modulus, there were few differences between the moduli of coupled and uncoupled GF composites at the same reinforcement contest, showing the above-mentioned limited effect of the interphase in the Young’s modulus. In any case, GF-based composites showed tensile strengths superior to CF-based materials. Only the PP + 50CF was superior to the PP + 20GFs. In the case of the Young’s modulus, CF showed a notable stiffening capability, and the composites. Composites with 40 wt % of CF showed a Young’s modulus similar to composites with a 20 wt % of GF, and so on.

**Figure 2:**
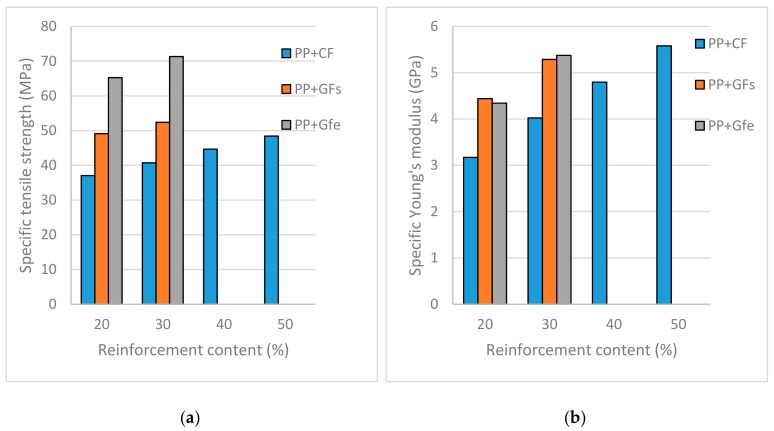
Specific tensile properties of curauá, and uncoupled and coupled glass fiber-reinforced polypropylene composites: (**a**) Specific tensile strength; (**b**) Specific Young’s modulus.

As commented upon in the introduction, one of the goals of the automotive industry is lightweighting. The densities of CF (Table 1) and GF (Table 2) composites show how at the same reinforcement contents, CF composites are lighter than GF, but only slowly. Figure 2 shows the specific tensile strength and Young’s modulus of the composites.

GF-based composites showed higher specific properties at the same reinforcement contents. Figure 2 show how only a composite with a 50 wt % CF content can reach more similar specific tensile strengths than an uncoupled GF composite with 20 wt % contents. In the case of the Young’s modulus, the, CF composites with 50 wt % contents showed values superior to any GF material.

Automotive components are usually designed to sustain reasonable deformation under use conditions. Thus, the stiffness of the composites can prove, to a limit, more important than its tensile strength when applied to a test case.

### 3.2. Test Case

#### 3.1.1. Car Interior Door Handle

**Figure 3:**
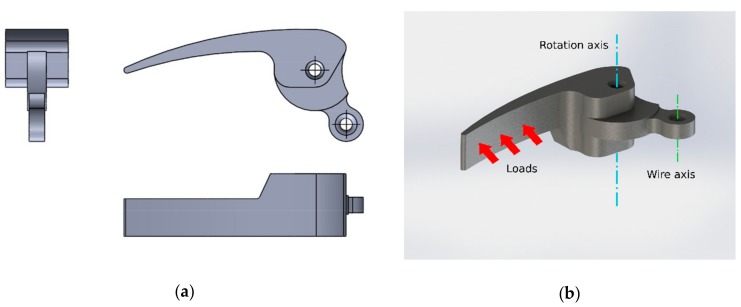
Digital mockup of the car interior door handle used as our test case: (**a**) Normalized views; (**b**) Perspective view.

The test case is a car interior door handle. This component was chosen because is a widely known mechanism, with few variations in its operating principle. Figure 3 shows different views of the modeled component.

A similar design was used by some of the authors in a recent study on the application of natural fiber-reinforced polyamide 11 composites [13]. The reference model was made of an uncoupled GF-reinforced PP composite, with a 20 wt % GF content. The mechanical properties of such material are shown in Table 2.

The operating principle of the mechanism is based on a class 1 lever. The fulcrum is the rotational axis, inserted in the handle and in the car door. The load is applied with the fingers in one side (Figure 3b). The resistance is placed in the wire axis, which conveys the loads to the opening mechanism. The wire is under tensile loads, ensuring the return of the mechanism to the designed neutral position.

The load needed to operate the mechanism was measured with a dynamometer on five car doors. The highest value was found to be 15 N. This value was multiplied by a 1.5 safety factor to stablish 20 N as a load, under which almost any car door handle can be operated under normal conditions. These loads are always exerted with one or two fingers and the thumbs. The literature shows that it is possible to exert up to 70 N loads with two fingers [47]. These two loads define the analyzed use conditions. The 20 N hypothesis defines a proper use of the mechanism, the 70 N a misuse. Nonetheless, both situations are possible, and must be taken into account in the following analysis.

#### 3.1.2. Analysis of the Use of CF-Reinforced PP Composites.

The loads and restrictions used to simulate the mechanism were:

- Loads were applied in the interior face of the lever (Figure 3b)
- The hole around the rotation axis was limited to a rotation degree of freedom, hindering all the other movements.
- The wire axis was limited in all of its degrees of freedom to simulate the reaction forces when the lever is loaded.

The analysis was defined as static. Then, the solid was meshed with standard quad point elements. A mesh of 8235 elements, with a mean size of 2.3 ± 0.11 mm, was created before some refining operations. Almost all of the elements (98.4%) showed aspect ratios below 3.

Table 1 and Table 2 show the mechanical properties used to perform the analyses. All the materials were applied to test the model under normal and misuse conditions. The results were collected in the shape of von Mises strengths (MPa), percentage displacements (%), net displacement (mm) and safety factors. The safety factor is defined as the ratio between the yield stress and the working stress. CF-based composites are not ductile materials, and yield and ultimate stresses are very similar.

**Figure 4:**
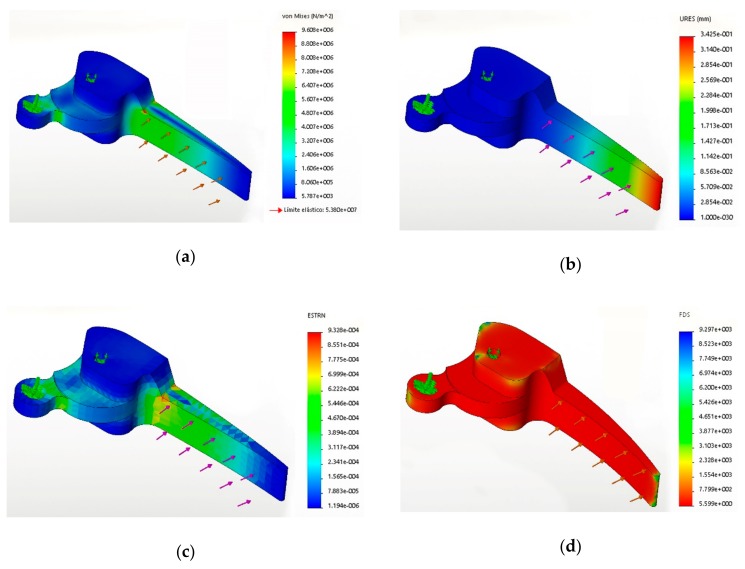
Graphical output obtained after assaying a component made of a 50 wt % CF-reinforced composite under 20 N loads: (**a**) Von Mises; (**b**) Net displacements; (**c**) Strain; (**d**) Safety factor.

Figure 4 shows the output provided by the analysis software for a component made of a CF-reinforced PP composite with a 50 wt % CF content.

The von Mises diagram obtained for all the studies was very similar, with only slight changes, as shows Table 3. The maximum von Mises stress was located at a zone were the area of the handle changes. This area coincides with an edge, a known stress raiser. It must be pointed out that the model submitted to analysis lacked some rounding operations that could mitigate such stress concentration but ease the meshing. Nonetheless, the maximum stresses were located at the expected areas. Thus, the analysis was considered accurate. The net displacements changed noticeably form one composite to the other, as expected due to the different Young’s moduli of such materials. The strains evolved similar to the displacements, and were always inferior to the stresses at break of the materials; thus no collapse was previewed. Safety factor diagrams showed a very regular coloring, mostly on the low safety factor area. This shows that the design is balanced, and no waste of material was done [13,22].

Table 3 shows the results obtained for the models under normal use conditions (20 N).

Under normal circumstances, the component must be far from its collapse or breaking point. This is shown by the obtained safety factors, all noticeably above 2. Nonetheless, normal use conditions must ensure that the deformation of the component does not compete with the developments of its function. The handle is submitted to a load that tends to deform the element. The maximum deformations are shown in Table 4 as net displacements. It was found that all the composite materials ensure deformations below 1mm, and pp matrix deformations around 1.4 mm. Any of these deformations endangers the handle function deployment, but affects the perceived quality of such component. Thus, having in account that the original component was made of PP + 20GFs, the net displacement of such composite was used as a reference. In order to avoid placing a too strict limit, a ±0.1 tolerance was proposed. Thus, all the materials that ensure net displacements lower that 0.5 mm were accepted as suitable as replacement materials.

Thus, all the GF-based materials can be used, and also, the CF-based composites, where CF contents from 30 wt % and above can be also candidates.

Table 4 shows the values obtained after submitting the model to misuse conditions (70 N).

The values increased in consonance with the increased load. All neat deformations were above 1 mm, and the safety factors decreased noticeably. In fact, a car handle made of PP was unable to endure the loads, and the analysis previews a breakage of the element. The rest of these materials returned a safety factor above 1. Alike the normal use conditions case, in the misuse hypothesis, the value obtained for the PP + 20GFs composite was used as a reference. In this case, a component was ruled suitable if it showed safety factors around 1.5. A similar tolerance to the applied for normal use conditions was applied to the safety factor, allowing all the components with safety factors above 1.4. Thus, all the GF-based composites fulfill the condition, and CF-based materials with 40 wt % or 50 wt % reinforcement contest returned favorable values.

**Figure 5:**
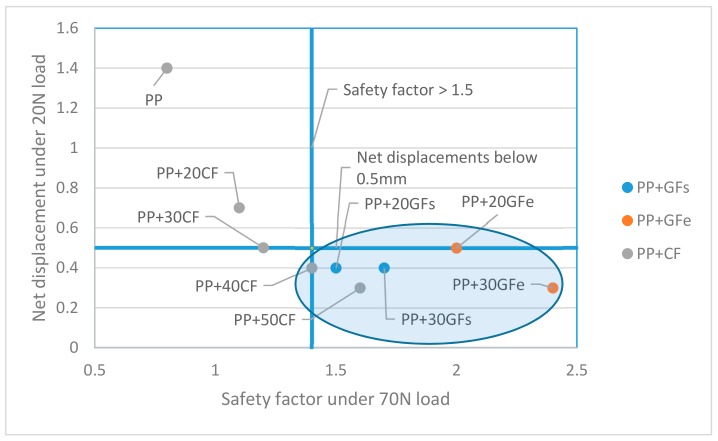
Combined requirements for the normal use and misuse conditions. The ellipse encircles the suitable materials that fulfill both requirements.

Two criteria were defined: On the one hand, the components under normal use conditions must show maximum displacements below 0.5 mm. On the other hand, the handles under misuse conditions must return safety factors above 1.5. Figure 5 shows the materials that fulfill both condition.

The figure shows how all the GF-based composites (uncoupled and coupled) fulfill the conditions necessary to be used for a car door handle. On the other hand, only the composites with 40 wt % and 50 wt % contents were able to substitute the original GF-based material.

The mentioned criteria were based on the mechanical properties of the materials. Nonetheless, other criteria, like lightweighting and environmental impact, must be taken into account.

Table 3 shows the mass of the components. The densities of GF and CF are 2.46 and 1.44 g/cm^3^, respectively. These densities are higher than the matrix (0.905 g/cm^3^); thus, adding reinforcement contents increased the density of the composites and the mass of the handles. The reference model shows a mass of 11.7 g. The handle with a 30 wt % of GF shows a 7.7% weight increase. The materials adding a 40 and 50 wt % of CF increased the weight of the reference models by 2.6% and 7.2%. Thus, replacing the GF by CF increased the weight of the components and disagreed with the lightweighting criteria. Notwithstanding, the differences are slight, and the environmental impact criteria must be considered.

The environmental impact analysis was performed under the following conditions:

- Manufacturing process, injection molding
- The elements are manufactured in Europe, to be consumed in Europe
- The lifespan is of 15 years
- At the end of life only 5% of the total is dumped

Table 5 shows the results of the preliminary LCA analysis. The analysis was performed only for the composites suitable to substitute the original PP + 20GFs composite. The analysis does not distinguish between coupled and uncoupled GF-based materials because the database lacks information on the environmental impact of the coupling agents. The environmental impacts of a fully PP component were added as control.

**Figure 6:**
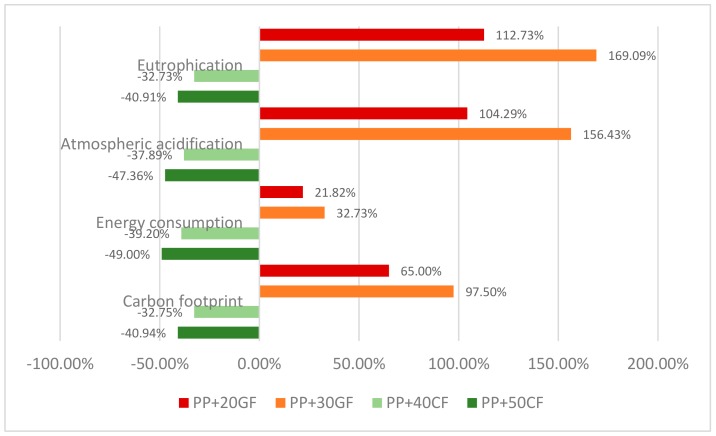
Percentage differences between the environmental impact of a polypropylene (PP) component and curauá mechanical pulp (CF)- and glass fiber (GF)-based composites.

The results show how the impact increases noticeably when the percentage of GF is increased, but also shows how the same impact decreases fast when the percentage of CF increases. In order to compare the environmental impacts, Figure 6 shows the percentage increases and decreases against PP.

The figure clearly shows the impact of adding glass fibers to a composite. Adding 20 wt % and 30 wt % of GF increases 65% and 97% the carbon footprint of a PP-based component. On the other hand, including curauá fibers decreases the carbon footprint. This behavior is similar for all the other considered impacts. Thus, though CF-based composites do not lightweight the door handle, they do contribute noticeably to decrease its environmental impact. As cited in the introduction, alternative materials must show technical, economic and environmental performances [2]. Curauá-reinforced PP composites showed technical performance equal to GF-reinforced PP composites, and better environmental behavior. The economic performance is out of the scope of the article, and further research is needed to compare the costs between CF- and GF-reinforced materials. Nonetheless, if these natural fibers-based composites are adopted by the automotive industry, surely industrial partners that will produce such composite pellets will provide such materials. Although the mixing equipment used to produce the composites is an industrial scale equipment, other processes like twin extruders can be used, and this can affect the costs, the properties of the materials (due to the morphology of the fibers) and the environmental impact.

## 4. Conclusions

Coupled Curauá fiber-reinforced polypropylene materials were formulated, mixed and textile tested. The linear evolution of the tensile strength and the Young’s modulus of the composites against reinforcement content indicated a good dispersion of the reinforcements and the presence of chemical interactions in the interphase. The composites added 20 wt % to 50 wt % reinforcement contents.

The tensile properties of the curauà-based composites were similar to uncoupled glass fiber-based composites with 20 wt % less reinforcement contents.

A car interior door handle was proposed as the test case to assess the possibility to change form a glass fiber-reinforced polypropylene composite to a curauá fiber-reinforced material. Normal use and misuse conditions were defined, and a finite element analysis of the test case was performed. The results showed that it was possible to replace an uncoupled 20 wt % glass fiber-reinforced polypropylene by a 40 wt % or 50 wt % curauà-reinforced material.

Curauá-based materials able to replace glass fiber showed higher densities. Thus, it was not possible to lightweight the component by changing from GF to CF.

The environmental impact of CF-based composites was noticeably lower than GF-based materials. From an environmental point of view, changing from mineral reinforcements to curauá fibers makes sense. Nonetheless, a more accurate life cycle analysis is needed to explore the sensitivity of the environmental impact to all the possible variables.

A micromechanics analysis of the interphase is needed to assess the strength of such interphases. From this information it is possible to identify whether or not it is also possible to further increase the mechanical properties of curauá fibers-reinforced materials.

More research is needed to evaluate the effects of water absorption on the mechanical properties of the composites.

## Figures and Tables

**Table 1:** Tensile properties of coupled curauá-reinforced polypropylene composites against reinforcement contents.

Sample	V^F^	*ρ^C^* (g/cm^3^)	*σ_t_^C^* (MPa)	*E_t_^C^* (GPa)	ε_t_^C^ (%)
PP	0	0.905	27.6 ± 0.5	1.5 ± 0.1	9.3 ^1^ ± 0.2
PP + 20CF	0.136	0.977	36.2 ± 0.6	3.1 ± 0.1	2.8 ± 0.1
PP + 30CF	0.212	1.019	41.5 ± 0.7	4.1 ± 0.1	2.3 ± 0.1
PP + 40CF	0.295	1.063	47.5 ± 0.8	5.1 ± 0.2	2.1 ± 0.1
PP + 50CF	0.386	1.111	53.8 ± 1.2	6.2 ± 0.2	1.9 ± 0.1

^1^ This is the strain at maximum strength.

**Table 2:** Tensile properties of uncoupled and coupled glass fiber-reinforced polypropylene composites against reinforcement contents.

Sample	V^F^	*ρ^C^* (g/cm^3^)	*σ_t_^C^* (MPa)	*E_t_^C^* (GPa)	ε_t_^C^ (%)
PP + 20GFs	0.084	1.036	50.9 ±4.3	4.6 ± 0.1	3.1 ± 0.1
PP + 30GFs	0.136	1.116	58.5 ± 4.3	5.9 ± 0.2	3.0 ± 0.2
PP + 20GFe	0.084	1.036	67.6 ± 0.9	4.5 ± 0.2	4.7 ± 0.2
PP + 30GFe	0.136	1.116	79.6 ± 1.2	6.0 ± 0.1	4.4 ± 0.2

**Table 3:** Main outputs of the analysis of the test case under 20 N loads.

Sample	Safety Factor	Net Displacement (mm)	Percentage Displacement (%)	Von Mises (MPa)	Mass (g)
PP	2.9	1.4	0.4	9.6	10.2
PP + 20GFs	5.3	0.4	0.1	9.6	11.7
PP + 30GFs	6.1	0.4	0.1	9.6	12.6
PP + 20GFe	7.0	0.5	0.1	9.6	11.7
PP + 30GFe	8.3	0.3	0.1	9.6	12.6
PP + 20CF	3.8	0.7	0.2	9.6	11.0
PP + 30CF	4.3	0.5	0.1	9.6	11.5
PP + 40CF	4.9	0.4	0.1	9.6	12.0
PP + 50CF	5.6	0.3	0.1	9.6	12.5

**Table 4:** Main outputs of the analysis of the test case under 70 N loads.

Sample	Safety Factor	Net Displacement (mm)	Percentage Displacement (%)	Von Mises (MPa)
PP	0.8	5.0	1.3	33.7
PP + 20GFs	1.5	1.6	0.4	33.7
PP + 30GFs	1.7	1.3	0.3	33.7
PP + 20GFe	2.0	1.7	0.5	33.7
PP + 30GFe	2.4	1.2	0.3	33.7
PP + 20CF	1.1	2.4	0.7	33.7
PP + 30CF	1.2	1.8	0.5	33.7
PP + 40CF	1.4	1.4	0.4	33.7
PP + 50CF	1.6	1.2	0.3	33.7

**Table 5:** LCA analysis of a door car handle made with the considered materials.

Sample	Carbon Footprint (kg CO_2_)	Energy Consumption (MJ)	Atmospheric Acidification (kg SO_2_)	Eutrophication (kg PO_4_)
PP	0.048	1.10	1.40 × 10^−4^	1.10 × 10^−5^
PP + 20GF	0.079	1.34	2.86 × 10^−4^	2.34 × 10^−5^
PP + 30GF	0.095	1.46	3.59 × 10^−4^	2.96 × 10^−5^
PP + 40CF	0.032	0.67	8.70 × 10^−4^	7.40 × 10^−6^
PP + 50CF	0.028	0.56	7.37 × 10^−4^	6.50 × 10^−6^
